# Development of C646‐Based Proteolysis Targeting Chimeras Degraders of the Lysine Acetyltransferases CBP and p300

**DOI:** 10.1002/cmdc.202400792

**Published:** 2025-04-11

**Authors:** Francesco Fiorentino, Filippo Spriano, Daniela Tomaselli, Giorgia Risi, Emanuele Fabbrizi, Valeria Pecci, Simona Nanni, Francesco Bertoni, Dante Rotili, Antonello Mai

**Affiliations:** ^1^ Department of Drug Chemistry and Technologies Sapienza University of Rome P.le A. Moro 5 00185 Rome Italy; ^2^ Faculty of Biomedical Sciences Institute of Oncology Research USI Bellinzona 6500 Bellinzona Switzerland; ^3^ Department of Translational Medicine and Surgery Università Cattolica del Sacro Cuore 00168 Rome Italy; ^4^ Fondazione “Policlinico Universitario A. Gemelli IRCCS” 00168 Rome Italy; ^5^ Oncology Institute of Southern Switzerland (IOSI) Ente Ospedaliero Cantonale Bellinzona 6500 Bellinzona Switzerland; ^6^ Department of Science “Roma Tre” University V.le G. Marconi 446 00146 Rome Italy; ^7^ Biostructures and Biosystems National Institute (INBB) Via dei Carpegna 19 00165 Rome Italy

**Keywords:** acetylation, epigenetics, lymphoma, proteolysis targeting chimeras, targeted degradation

## Abstract

The alteration of the lysine acetyltransferase activity and protein–protein interactions of the transcriptional co‐activators CREB‐binding protein (CBP) and p300 is linked to the development of both solid and hematological cancers. To target both functions of CBP/p300, two PROTAC‐based chemical degraders are developed by linking the CBP/p300 catalytic inhibitor C646 and the Cereblon (CRBN) ligand thalidomide via polyethylene glycol‐based linkers. Both compounds exhibit submicromolar inhibition of CBP/p300 and decrease their levels in the SU‐DHL‐10 lymphoma cell line at low‐micromolar concentrations. Moreover, it is demonstrated that compound **1** recruits CBP/p300 and CRBN in cells and acts as a bona fide PROTAC degrader of CBP/p300 via the ubiquitin‐proteasome pathway. Finally, both compounds exhibit low‐micromolar antiproliferative activity in different lymphoma cell lines and are more potent than C646. Overall, it is demonstrated that the PROTAC strategy is a viable option for targeting CBP/p300 in lymphoma and identifies compound **1** as a promising chemical tool and lead compound for further studies.

## Graphical abstract

Two PROTAC‐based degraders targeting the cancer‐associated transcriptional co‐activators CBP/p300 weredeveloped. These degraders, combining the CBP/p300 inhibitor C646 with the CRBN ligand thalidomide, reduced CBP/p300 levels in lymphoma cells at low‐micromolar concentration. Compound **1** degrades CBP/p300 via the ubiquitin‐proteasome pathway and exhibits low‐micromolar antiproliferative activity. These data highlight the PROTAC strategy as a promising approach for targeting CBP/p300 in cancer .

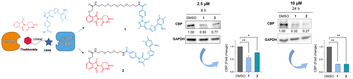

## 1 Introduction

The lysine acetyltransferases CREB‐binding protein (CBP, KAT3A) and p300 (KAT3B) are key factors involved in transcriptional activation and share high sequence homology along with overlapping functions.^[^ ^1^ ^]^ Both proteins have been shown to acetylate Lys27 on histone H3 and, in cooperation with the acetyl reader BRD4, support the transcription process.^[^ ^2^ ^]^ Nevertheless, the two proteins also target other non‐histone proteins such as p53, NF‐κB, c‐Myc, Foxo1, STAT3, and β‐catenin.^[^ ^1^ ^]^ Furthermore, CBP and p300 exert their functions by interacting with different proteins, including chromatin regulators and DNA‐binding proteins.[^1^, ^3^] Indeed, both CBP and p300 are multidomain proteins, and beyond the catalytic domain, they possess a bromodomain (BD), an *N*‐terminal nuclear receptor interaction domain (NRID), four zinc finger domains (TAZ1, PHD, ZZ, and TAZ2), and a kinase‐inducible domain interacting (KIX) domain.^[^ ^4^ ^]^ In line with this, the interactome of CBP and p300 has been indicated to comprise roughly 400 proteins,^[^ ^1^, ^3^ ^]^ thus suggesting that they both exert their function *via* catalytic and scaffolding roles.

Because of the extensive biochemical processes associated with their activities and their near‐ubiquitous distribution in tissues, these proteins serve as important hubs for various cellular signaling pathways, including growth, proliferation, differentiation, development, and homeostasis. Given the manifold functions of CBP and p300, the alteration of their activity has been connected to the onset and development of different cancer types.^[^ ^1^, ^5^ ^]^ Different forms of cancer, including both solid tumors and hematological cancers depend on CBP and p300 activity.^[^ ^6^ ^]^ Moreover, the *CREBBP* and *EP300* genes, coding for CBP and p300, respectively, are often deleted or mutated in various cancers, especially in lymphomas, leading to the overexpression of genes supporting tumor growth.^[^ ^7^ ^]^ Mutations or deletions are predominantly monoallelic, with the remaining normal copy still expressed, and events inactivating CBP and p300 are mutually exclusive, indicating that tumor cells necessitate at least one of the two proteins.[^6^, ^7^] These data have prompted various research groups to work on developing CBP/p300 inhibitors to be used as both potential therapeutics and chemical tools to scrutinize CBP and p300 biology.[^1^, ^8^] These include the pyrazolone derivative C646[^8^, ^9^] and the recently reported oxazolidinedione A‐485^[^ ^10^ ^]^ and its analog B026.^[^ ^11^ ^]^ More recently, inhibitors of the BD domain of CBP and p300 have been disclosed (GNE‐207,^[^ ^12^ ^]^ GNE‐781,^[^ ^13^ ^]^ and CCS1477/ inobrodib^[^ ^14^ ^]^), with CCS1477 advancing toward clinical stage for both hematological and solid tumors.^[^ ^14^ ^]^ Nevertheless, since these proteins have both catalytic and scaffolding activities, targeting either one of them may result in only a partial suppression of their carcinogenic function. Hence, a desirable approach would tackle both catalytic and scaffolding activities of CBP and p300. Indeed, the combination of the p300/CBP catalytic inhibitor A‐485 and the BD inhibitor I‐CBP112 in a panel of prostate cancer cells has shown synergistic activity in inhibiting both p300 chromatin occupancy and cancer cell proliferation.^[^ ^15^ ^]^

Another viable approach to abolishing both the scaffolding and catalytic roles of CBP and p300 may consist of developing chemical degraders that would induce the proteasome‐mediated degradation of the target proteins.^[^ ^16^ ^]^ To this end, research efforts have been made toward developing proteolysis targeting chimeras (PROTACs) to impair CBP and p300 cellular functions by promoting their degradation. PROTACs are heterobifunctional molecules constituted by an E3 ligase recruiting moiety connected to a warhead that binds the protein of interest (POI) *via* a linker. PROTACs bring an E3 ligase and a POI into close contact, inducing poly‐ubiquitination and proteasomal degradation of the POI, and are subsequently released and recycled to induce the degradation of a new POI, thus presenting a catalytic mechanism of action.^[^ ^17^ ^]^ Compared to classical inhibitors, they usually show higher potency and selectivity, prolonged pharmacodynamic effects, and the ability to evade most resistance mechanisms based on target mutations.^[^ ^17^ ^]^ To date, different PROTACs have been developed for many targets, some reaching the clinical arena. In the case of CBP and p300, a few PROTACs have been developed using different p300/CBP BD inhibitors as warheads,^[^ ^18^ ^]^ with the recently developed CBPD‐268 inducing CBP/p300 degradation at nanomolar doses and showing promising in vivo activity in prostate cancer mouse models.^[18d]^ Furthermore, the catalytic inhibitor A‐485 was also used as a warhead in JQAD1, a CBP/p300‐directed PROTAC shown to decrease neuroblastoma cell viability with IC_50_ values in the nanomolar range (Figure S1, Supporting Information).^[^ ^19^ ^]^ All the reported PROTACs possess E3 ligase ligands capable of recruiting the E3 ligase Cereblon (CRBN).^[^ ^20^ ^]^

Given the potential of targeting CBP and p300 activity through targeted degradation, we set out to develop a new class of PROTAC degraders. We based our design on the catalytic inhibitor C646, shown to be active against different solid and hematological cancers,[^8^, ^9^] as a warhead and chose thalidomide as a CRBN‐recruiting moiety. We designed and synthesized two PROTACs (compounds **1** and **2**) and evaluated them in terms of CBP and p300 inhibition. We then assessed the two compounds in cellular models of diffuse large B‐cell lymphoma (DLBCL), in which a synthetic lethal interaction between CBP and p300 has been described.[^7^, ^21^] Inhibition of CBP catalytic activity resulted in decreased cancer cell viability.[^7^, ^10^] Nevertheless, given the involvement of both the catalytic and scaffolding roles of CBP and p300 in promoting cancer cell growth, abolishing both of them *via* targeted degradation may represent a successful strategy.

## 2 Results and Discussion

### 2.1 Chemistry

**Figure 1:**
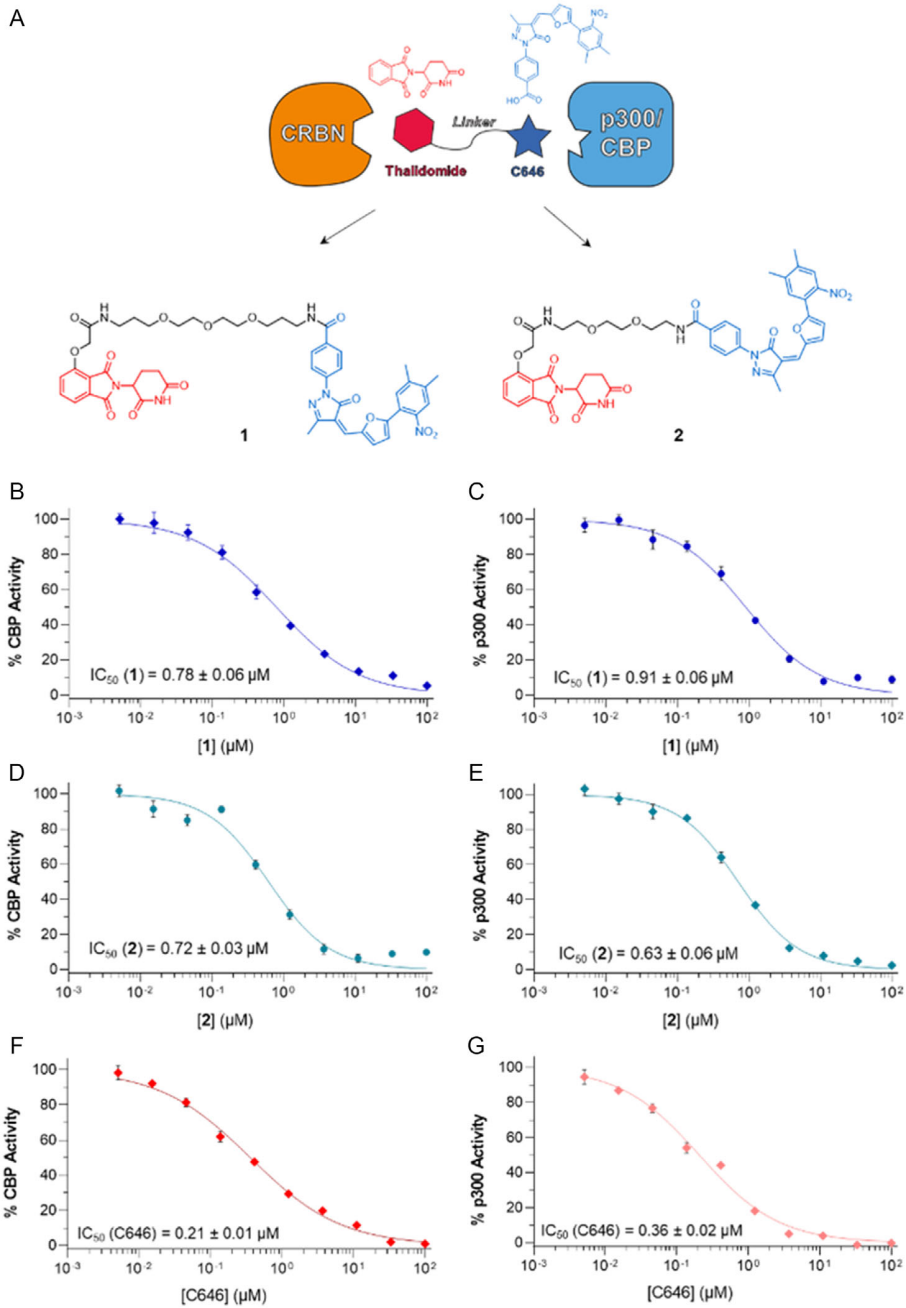
A) Design of compounds **1** and **2** based on C646 and thalidomide structures. B,C) Dose–response curves of CBP (B) and p300 (C) catalytic activity as a function of compound **1** concentration. D,E) Dose–response curves of CBP (D) and p300 (E) catalytic activity as a function of compound **2** concentration. F,G) Dose–response curves of CBP (F) and p300 (G) catalytic activity as a function of C646 concentration. Values are means ± standard deviation (S. D.) of three independent experiments (*n* = 3).

We designed compounds **1** and **2** by linking the structures of the known p300/CBP catalytic inhibitor C646 (**3**) with the CRBN ligand thalidomide through two types of polyethylene glycol (PEG)‐based linkers: an *N*‐(3‐(2‐(2‐(3‐aminopropoxy)ethoxy)ethoxy)propyl)‐2‐oxyacetamide linker for compound **1** and a shorter *N*‐(2‐(2‐(2‐aminoethoxy)ethoxy)ethyl)‐2‐oxyacetamide linker for compound **2** (**Figure** **1**A).

**Scheme 1:**
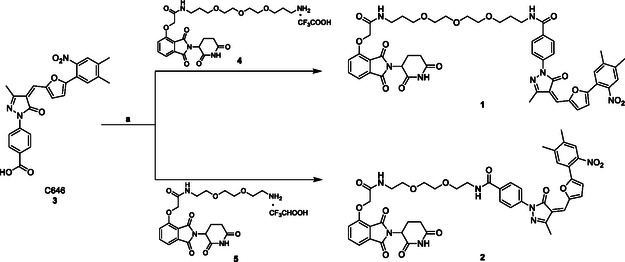
Synthesis of compounds **1** and **2**. *Reagents and conditions:* a) HATU, DIPEA, dry THF, N_2_, rt, 6 h.

The synthesis of compounds **1** and **2** is depicted in **Scheme** **1**. Both molecules were prepared through a coupling reaction between C646 (**3**), prepared as previously reported,^[9a]^ and the appropriate linker‐substituted thalidomide derivatives synthesized as trifluoroacetic salts **4** ^[^ ^22^ ^]^ and **5** ^[^ ^22^, ^23^ ^]^ as reported in literature. Briefly, C646 was mixed with either **4** or **5** at room temperature (rt) under a nitrogen atmosphere in dry THF using hexafluorophosphate azabenzotriazole tetramethyluronium (HATU) as a coupling reagent and *N,N*‐diisopropylethylamine (DIPEA) as a base (Scheme 1), finally affording the desired compounds **1** and **2**.

### 2.2 Biochemical Characterization of Compounds 1 and 2

Although based on the structure of C646, we wondered whether adding the CRBN‐recruiting moiety could alter the capability of compounds **1** and **2** to engage the target proteins. To answer this question, we initially characterized them both for their inhibition of CBP and p300 catalytic activities. Our assays indicated that both derivatives **1** and **2** retained their inhibitory activity toward CBP and p300, with IC_50_ values ranging from 0.6 to 0.9 μM (Figure 1B–E). Nevertheless, adding the CRBN‐recruiting moiety reduced their potency compared to C646 by a factor of 2 to 4, suggesting a slight hindrance in their binding (Figure 1B–G).

### 2.3 Evaluation of the Mode of Action of **1** and **2** in DLBCL Cells

We then evaluated the capability of compounds **1** and **2** to induce the degradation of the target proteins in the DLBCL cell line SU‐DHL‐10.

**Figure 2:**
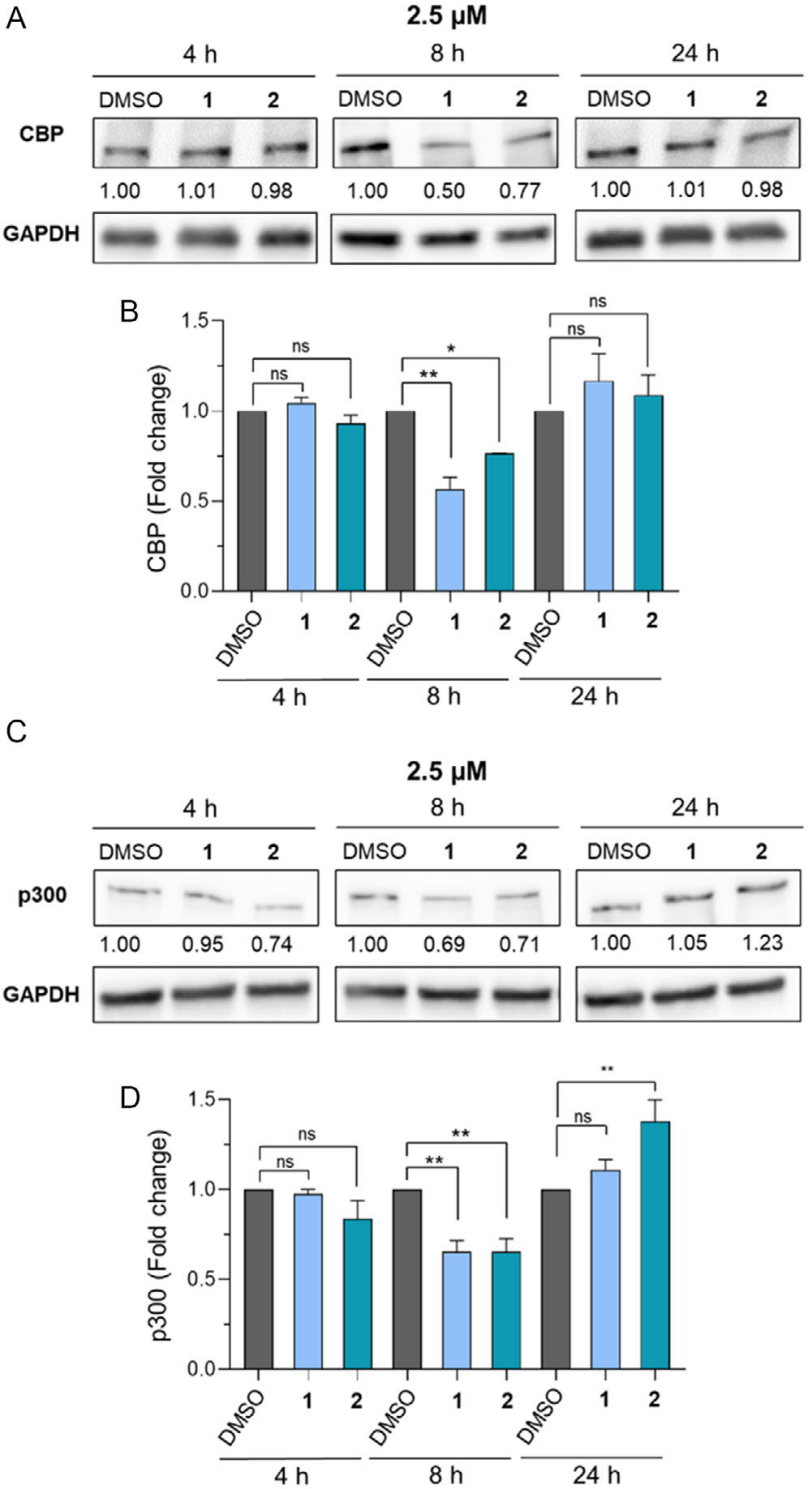
A) WB analysis of CBP levels in the DLBCL SU‐DHL‐10 cell line exposed to compounds **1** and **2** (2.5 μM) for 4, 8, and 24 h. GAPDH has been used as a loading control. B) Quantification of CBP levels by densitometric analysis. C) WB analysis of p300 levels in the SU‐DHL‐10 cell line exposed to compounds **1** and **2** (2.5 μM) for 4, 8, and 24 h. GAPDH has been used as a loading control. D) Quantification of p300 levels by densitometric analysis. The relative protein levels are expressed as a fold change of treated versus untreated samples, after GAPDH normalization. Experiments were performed in duplicate (*n* = 2). At each time point, the statistical analysis compares compound treatment versus control (ns, nonsignificant; *, p < 0.05; **, p < 0.01; ***, p < 0.001; Student's *t*‐test). Control (DMSO) consists of 1% (v v^−1^) DMSO‐treated cells.

Western blot (WB) experiments indicated that, when tested at a concentration of 2.5 μM, compound **1** reduced the levels of CBP following 8 h treatment, while **2** was slightly less active (**Figure** **2**A,B). Moreover, protein levels returned to the initial levels as the control after 24 h (Figure 2A,B). This was most likely a consequence of restored protein synthesis during the time frame of the experiment. We observed a similar pattern in the case of p300, whereby both compounds decreased protein levels after 8 h incubation, while no difference was observed after 24 h treatment (Figure 2C,D).

**Figure 3:**
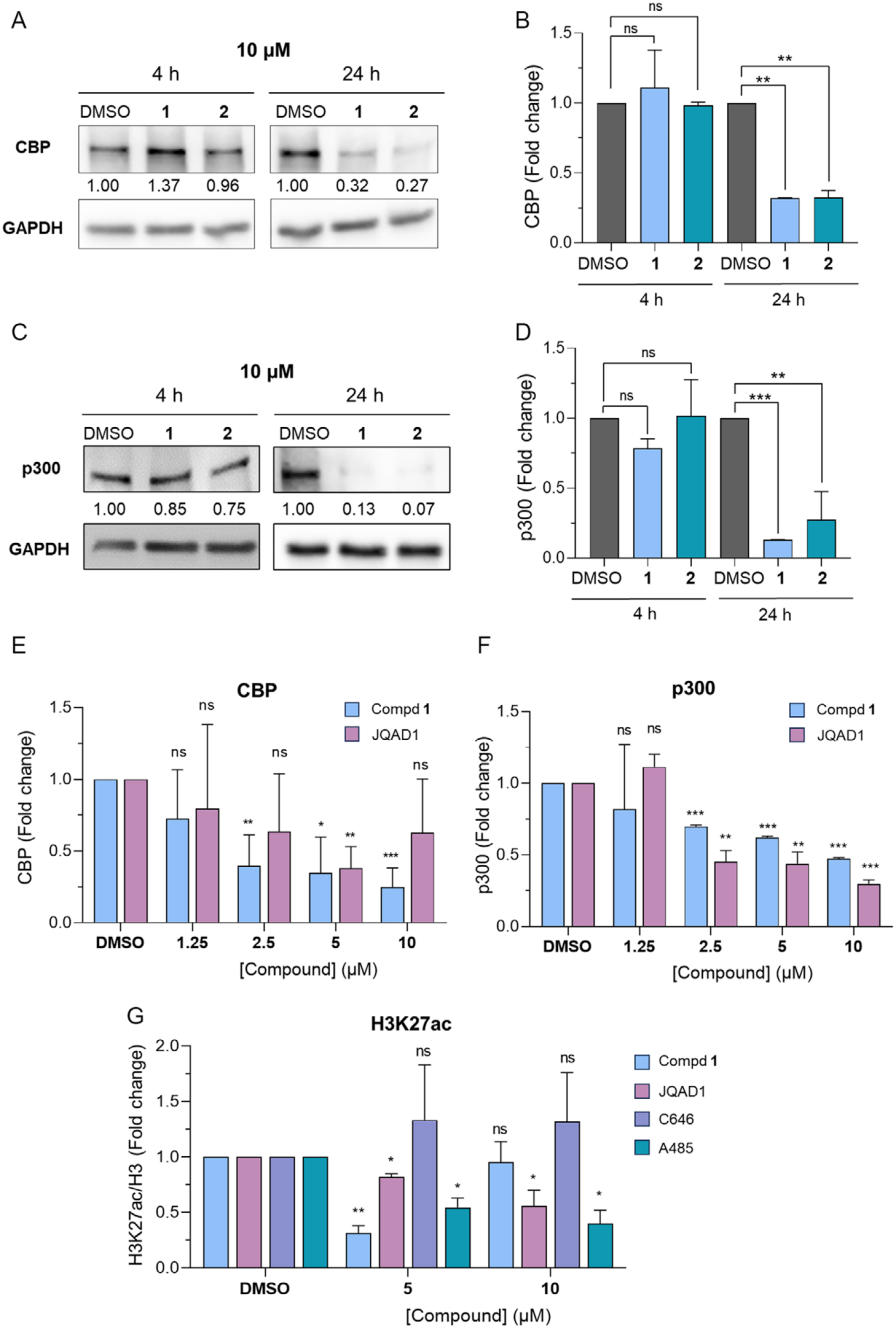
A) WB analysis of CBP levels in the SU‐DHL‐10 cell line exposed to compounds **1** and **2** (10 μM) for 4 and 24 h. GAPDH has been used as a loading control. B) Quantification of CBP levels by densitometric analysis. C) WB analysis of p300 levels in the SU‐DHL‐10 cell line exposed to compounds 1 and 2 (10 μM) for 4 and 24 h. GAPDH has been used as a loading control. D) Quantification of p300 levels by densitometric analysis. E) Quantification of CBP levels in the SU‐DHL‐10 cell line exposed to increasing concentrations of compound **1** and JQAD1 for 24 h. F) Quantification of p300 levels in the SU‐DHL‐10 cell line exposed to increasing concentrations of compound **1** and JQAD1 for 24 h. G) Quantification of H3K27ac levels in the SU‐DHL‐10 cell line exposed to compound **1**, JQAD1, C646, and A485 (5 and 10 μM) for 24 h. The relative protein levels are expressed as a fold change of treated versus untreated samples, after GAPDH normalization. Example WB images of (E–G) panels are in Figure S2, Supporting Information. Experiments were performed at least in duplicate (*n* = 2–3). At each time point, the statistical analysis compares compound treatment vs control (ns, nonsignificant; *, p < 0.05; **, p < 0.01; ***, p < 0.001; Student's *t*‐test). Control (DMSO) consists of 1% v/v DMSO‐treated cells.

Notably, when we increased the concentration of each compound to 10 μM, we induced protein degradation even after 24 h, with both compounds decreasing protein levels by roughly 70% (**Figure** **3**A,B). Analogously, both **1** and **2** significantly reduced p300 levels when administered at 10 μM for 24 h (Figure 3C,D).

Moreover, compound **1** demonstrated a dose‐dependent reduction in CBP and p300 protein levels after 24 h of treatment. It exhibited slightly better efficacy on CBP compared to the previously reported PROTAC JQAD1, while both compounds had a similar impact on p300, with JQAD1 displaying slightly higher potency (Figure 3E,F and S2A, B, Supporting Information). These findings align with previous reports indicating that JQAD1 is significantly more active toward p300 than CBP.^[^ ^19^ ^]^ Both compound **1** and JQAD1, along with the compounds corresponding to their target‐engaging warheads (C646 and A485), were evaluated for their effects on H3K27 acetylation, one of the primary targets of CBP and p300,[^1^, ^5^] after a 24 h incubation at concentrations of 5 and 10 μM (Figure 3G and S2C, Supporting Information). Interestingly, while JQAD1 and A485 reduced H3K27 acetylation in a dose‐dependent fashion, compound **1** decreased H3K27 acetylation at 5 μM, but not at 10 μM, and C646 did not reduce acetylation but instead caused a slight, albeit non‐statistically significant, increase at both concentrations. This apparently paradox effect can be attributed to the known capability of C646 to inhibit histone deacetylases (HDACs) at low micromolar concentrations.^[^ ^24^ ^]^ While this effect is evident for C646 at both tested concentrations, it only starts to become apparent for the C646‐based PROTAC **1** at higher concentrations. Additionally, the potential thiol reactivity of C646^[^ ^25^ ^]^ could contribute to non‐specific interactions, further influencing the effects observed with **1**. Nonetheless, the C646‐based PROTAC **1** induced a dose‐dependent reduction of both CBP and p300 levels, along with a decrease in H3K27 acetylation at 5 μM, suggesting the effective engagement of both proteins in cells.

**Figure 4:**
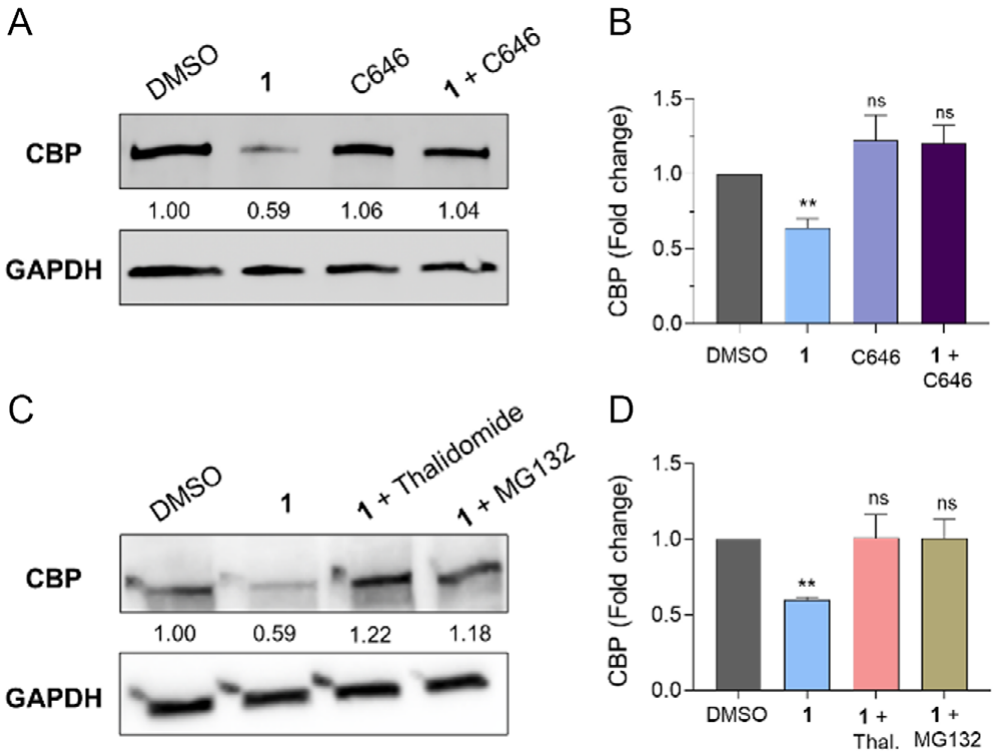
A) WB analysis of CBP levels in the DLBCL SU‐DHL‐10 cell line exposed to compound **1** (2.5 μM), C646 (10 μM), and their combination for 8 h. GAPDH has been used as a loading control. B) Quantification of CBP levels by densitometric analysis. The relative protein levels are expressed as a fold change of treated versus untreated samples, after GAPDH normalization. Experiments were performed in triplicate (*n* = 3). The statistical analysis compares compound treatment versus control (ns, nonsignificant; **, p < 0.01; one‐way ANOVA). Control (DMSO) consists of 1% (v v^−1^) DMSO‐treated cells. C) WB analysis of CBP levels in the DLBCL SU‐DHL‐10 cell line exposed to compound **1** (2.5 μM), a **1**/thalidomide combination ([**1**] = 2.5 μM; [thalidomide] = 10 μM), and a **1**/MG132 combination ([**1**] = 2.5 μM; [MG132] = 10 μM) for 8 h. GAPDH has been used as a loading control. D) Quantification of CBP levels by densitometric analysis. The relative protein levels are expressed as a fold change of treated versus untreated samples, after GAPDH normalization. Experiments were performed in triplicate (*n* = 3). The statistical analysis compares compound treatment vs control (ns, nonsignificant; **, p < 0.01; Student's *t*‐test). Control (DMSO) consists of 1% v/v DMSO‐treated cells.

To further investigate the mechanism of CBP/p300 degradation induced by our compounds, we performed WB‐based blocking experiments using compound **1**, which was more potent at 2.5 μM, focusing on CBP. We compared the CBP protein levels after 8 h incubation with either **1** (2.5 μM), C646 (10 μM) or the **1**/C646 combination. Compound **1** alone decreased the levels of CBP, while C646 had no effect on CBP levels (**Figure** **4**A,B). Notably, incubating the cells with the **1**/C646 combination also did not affect CBP levels (Figure 4A,B), thereby suggesting that C646 blocks the compound‐induced degradation by competing for its active site.

We also assessed whether co‐incubation of **1** with thalidomide (10 μM) or the proteasome inhibitor MG132 (10 μM) could reverse the effects observed in the presence of compound **1** alone by restoring the initial protein levels (Figure 4C,D). Hence, similar to what was observed with C646, compound **1** competed with thalidomide, albeit in this case, the competition was for the interaction with the E3 ligase CRBN. Moreover, proteasome inhibition abolishes **1**‐induced CBP degradation, thus indicating a proteasome‐dependent mechanism for the observed protein degradation. Overall, our blocking experiments indicate that **1** triggered the ubiquitin/proteasome‐mediated degradation of CBP, thereby acting as a *bona fide* CBP/p300‐targeting PROTAC.

### 2.4 Antiproliferative Activity of Compounds **1** and **2** in DLBCL Cells

**Figure 5:**
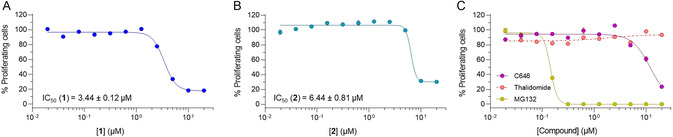
Antiproliferative activities of compounds **1 **A), **2** B), C646, thalidomide, and MG132 C) tested at increasing concentrations (0 to 20 μM) in the DLBCL SU‐DHL‐10 cells for 24 h. Control (0 μM) consists of DMSO‐treated cells.

Finally, we assessed the influence of **1** and **2** on the DLBCL SU‐DHL‐10 cell viability and compared their effects with those of C646, thalidomide, and MG132 (**Figure** **5**). We exposed the cells for 24 h to increasing doses (up to 20 μM) of each compound through the 3‐(4,5‐dimethylthiazol‐2‐yl)‐2,5‐diphenyltetrazolium bromide (MTT)‐based colorimetric assay. Both compounds exhibited dose‐dependent antiproliferative activity in SU‐DHL‐10 cells, with IC_50_ values of 3.44 and 6.44 μM, respectively. Notably, both derivatives were more potent than the parent compound C646, which displayed an IC_50_ value of 12.15 μM (Figure 5 and **Table** **1**), while thalidomide had no significant effects on cell viability. Overall, these findings suggest that the ability of the compounds to induce CBP/p300 proteasomal degradation has a significant contribution to their in vitro anti‐lymphoma activity.

**Table 1:** IC_50_ values (μM) of 1, 2, C646, thalidomide, and MG132 after 24 h treatment in SU‐DHL‐10 cells.^a)^

Compounda)	IC_50_ [μM]b)
**1**	3.44 ± 0.12
**2**	6.44 ± 0.81
C646	12.15 ± 3.02
Thalidomide	>20
MG132	0.144 ± 0.002

a) Values are means ± standard deviation (S.D.) of two separate experiments (*n* = 2).

b) Half maximal inhibitory concentration: dose required to reduce cell proliferation by 50%.

**Figure 6:**
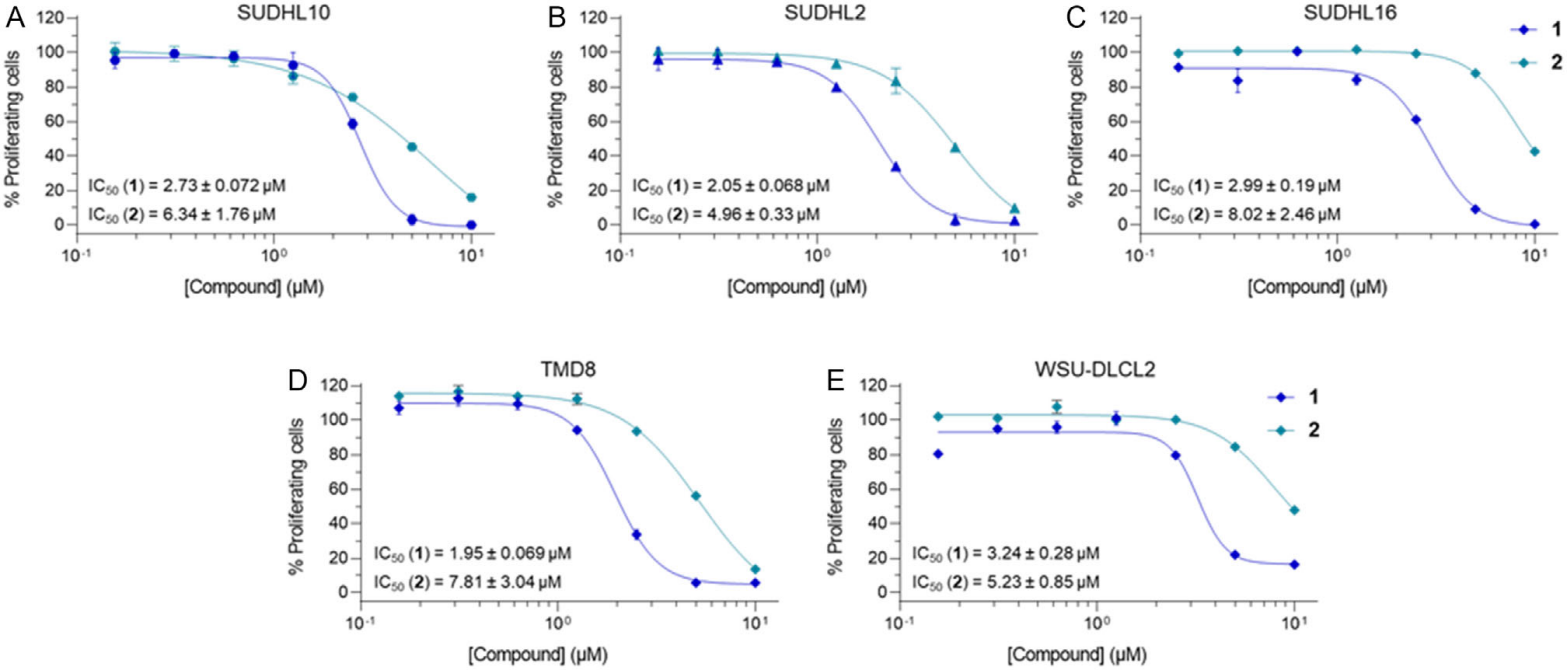
Antiproliferative activities of compounds **1** and **2** tested at increasing concentrations (0 to 10 μM) in A) SU‐DHL‐10, B) SU‐DHL‐2, C) SU‐DHL‐16, D) TMD8, and E) WSU‐DLCL2 cells for 72 h. Control (0 μM) consists of 1% (v v^−1^) DMSO‐treated cells.

To gain a better understanding of the anti‐lymphoma potential of compounds 1 and 2, we tested them in additional DLBCL cell lines, namely SU‐DHL‐2, SU‐DHL‐16, TMD8, and WSU‐DLCL2 (**Figure** **6**). We incubated these cell lines with either 1 or 2 at increasing concentrations for 72 h and measured their viability IC_50_ via the same MTT assay as mentioned earlier. The obtained IC_50_ values were in the range of 2–8 μM, with 1 being 2‐ to 4‐fold more potent than 2 (Figure 6 and Table S2, Supporting Information), in line with our previous observations.

## 3 Conclusion

CBP and p300 are crucial proteins regulating transcription and cell signaling through their catalytic activities, which target both histone and non‐histone proteins as well as protein–protein interactions. Through their catalytic activity, they are known to target roughly 5300 proteins^[^ ^26^ ^]^ and their interactome comprises more than 400 proteins.^[^ ^1^ ^,^ ^3a^ ^]^

The alteration of CBP and p300 activity is linked to the onset and development of different cancer types, including lymphomas.^[7e−j]^ Specifically, the development of DLBCL is supported by both the lysine acetyltransferase domain‐deficient p300 as well as a catalytically active CBP.[^7^, ^21^] This is an example of how both the catalytic and scaffolding functions trigger and support the oncogenic transformation. In line with this, inhibitors targeting both the catalytic domain and the BD domains of CBP and p300 have been developed, along with PROTACs.

In this study, we report the design, synthesis, in vitro and cellular evaluation of two PROTAC degraders based on the CBP/p300 catalytic inhibitor C646 by employing the CRBN ligand thalidomide as an E3 ligase recruiting moiety. Both compounds retained their inhibition towards CBP and p300 with IC_50_ values in the submicromolar range (Figure 1) and induced their degradation at both 2.5 μM (8 h incubation) and 10 μM (24 h incubation) (Figure 2 and 3A–D). Following a 24 h incubation, compound **1** caused dose‐dependent reduction of CBP and p300 levels, showing results comparable to those of the known PROTAC JQAD1, and could also reduce H3K27 acetylation at 5 μM (Figure 3E–G).

We then investigated their mode of action by focusing on compound **1**, showing that it functions as a *bona fide* PROTAC degrader of CBP/p300, acting by triggering the ubiquitin‐proteasome pathway. Indeed, C646 impaired compound **1**‐induced CBP degradation (Figure 4A). Moreover, when administered in the presence of an excess of the CRBN ligand thalidomide or the proteasome inhibitor MG132, compound **1** resulted inactive (Figure 4C,D). These findings indicate that compound **1** induces targeted protein degradation through the expected PROTAC mechanism.

Finally, the cell viability data showed that both compounds inhibited the proliferation of SU‐DHL‐10 and additional DLBCL cell lines with IC_50_ values in the low‐micromolar range (Figure 4 and 5, Table 1 and S2, Supporting Information). Compound **1** was slightly more active than **2**, in line with the CBP degradation observed at 2.5 μM. This may result from the longer linker that may facilitate the recruitment of the E3 ligase and the consequent formation of the ternary complex. Moreover, both **1** and **2** were more potent than C646 in SU‐DHL‐10 (Figure 5 and Table 1), thereby suggesting that CBP/p300 degradation is more effective than catalytic inhibition alone.

In summary, this study has led to the identification of two new CBP/p300 PROTAC degraders. Further optimization and investigation of the mode of action of compound **1** are needed to clarify its selectivity and enhance its potency and drug‐like properties. Notably, it contains structural moieties that may pose challenges in vivo such as the nitro group, while the presence of a Michael acceptor group has been associated with thiol reactivity and off‐target effects in its corresponding small molecule, C646. Nevertheless, our findings in DLBCL models suggest that inducing degradation of CBP/p300 proteins with compounds based on these structures could be an attractive strategy for lymphoma treatment. Additional research and structural modifications may lead to the development of chemical tools derived from these compounds, enabling the exploration of CBP and p300 functions in both physiological and pathological contexts.

## 4 Experimental Section

4.1

4.1.1

##### Chemistry: General

Melting points were determined on a Cole‐Parmer Stuart SMP20 digital melting point apparatus. ^1^H NMR spectra were recorded at 400 MHz on a Bruker AC 400 spectrometer, reporting chemical shifts in *δ* (ppm) units relative to the internal reference tetramethylsilane (Me_4_Si). All compounds were routinely checked by TLC and ^1^H‐NMR. TLC was performed on aluminum‐backed silica gel plates (Merck DC, Alufolien Kieselgel 60 F254) with spots visualized by UV light. Yields of all reactions refer to the purified products. All chemicals were purchased from Sigma‐Aldrich (Milan, Italy), and were of the highest purity. Mass spectra were recorded on an MSQ Plus Mass Spectrometer (Thermo Fisher), and samples were injected by a Harvard pump using a flow rate of 100 μL min^−1^, infused in the Electrospray system. All solvents were reagent grade and, when necessary, were purified and dried by standard methods. The concentration of solutions after reactions and extractions involved using a rotary evaporator operating at a reduced pressure of ≈20 Torr. Organic solutions were dried over anhydrous sodium sulfate. Elemental analysis has been used to determine the purity of all final compounds that is >95%. Analytical results are within ±0.40% of the theoretical values.

##### Chemistry: General Procedure for Synthesis of Final Compounds 1 and 2

DIPEA (45.13 mg, 0.349 mmol, 3.0 eq) and HATU (44.26 mg, 0.116 mmol, 1.0 eq) were added to a solution of C646 (51.85 mg, 0.116 mmol, 1.0 eq) and the appropriate trifluoroacetic salt of the linker‐substituted thalidomide (0.116 mmol, 1.0 eq) in dry THF (3.5 mL) and the solution was stirred at rt under a nitrogen atmosphere for 6 h. The reaction mixture was then evaporated under vacuum. The crude product was purified by two sequential silica gel chromatographic columns eluting with a chloroform/methanol (98:2) mixture for the first run and a chloroform/methanol/ammonia (40:1:0.1) mixture for the second run. The eluted product was then triturated with a dichloromethane/diethyl ether (1:8) mixture to afford the final compounds 1 and 2.

##### Chemistry: (Z)‐4‐(4‐((5‐(4,5‐dimethyl‐2‐nitrophenyl)furan‐2‐yl)methylene)‐3‐methyl‐5‐oxo‐4,5‐dihydro‐1 H‐pyrazol‐1‐yl)‐N‐(1‐((2‐(2,6‐dioxopiperidin‐3‐yl)‐1,3‐dioxoisoindolin‐4‐yl)oxy)‐2‐oxo‐7,10,13‐trioxa‐3‐azahexadecan‐16‐yl)benzamide (MC4385, 1)

Red solid; m.p.: 122–124 °C; recrystallization solvent: toluene; yield: 43%; ^1^H‐NMR (400 MHz, CDCl_3_) *δ* 1.77–1.83 (m, 4 H, NHCH_2_C*H*_2_CH_2_O + OCH_2_C*H*_2_CH_2_NH), 2.08 (m, 1 H, NCHC*H*HCH_2_CO glutarimide), 2.26 (s, 3 H, CH_3_), 2.32 (s, 3 H, CH_3_), 2.33 (s, 3 H, CH_3_), 2.64–2.84 (m, 3 H, NCHCH*H*C*H*_2_CO glutarimide), 3.38–3.60 (m, 16 H, 8 × CH_2_ linker protons), 4.53 (s, 2 H, OCH_2_CO), 4.87–4.89 (m, 1 H, NC*H*CH_2_CH_2_CO glutarimide), 6.85 (d, 1 H, C*H* furan proton), 7.06–7.13 (m, 2 H, CON*H* + C*H* phthalimide proton), 7.19 (s, 1 H, CH methine proton), 7.44–7.47 (m, 3 H, 2 × CH phthalimide protons + 1 × CH phenyl proton), 7.58 (s, 1 H, CH phenyl proton), 7.63 (s, 1 H, CON*H*), 7.80 (d, 2 H, 2 × CH benzamide protons), 8.00 (d, 2 H, 2 × CH benzamide protons), 8.68 (br s, 1 H, NH glutarimide), 8.74 (d, 1 H, CH furan proton). Elemental analysis for C_27_H_23_N_3_O_3_S: Calculated % = C, 62.49; H, 5.45; N, 8.75. Found % = C, 62.60; H, 5.46; N, 8.70. MS (ESI) m/z: 962 [M + H]^+^.

##### Chemistry: (Z)‐4‐(4‐((5‐(4,5‐dimethyl‐2‐nitrophenyl)furan‐2‐yl)methylene)‐3‐methyl‐5‐oxo‐4,5‐dihydro‐1 H‐pyrazol‐1‐yl)‐N‐(2‐(2‐(2‐(2‐((2‐(2,6‐dioxopiperidin‐3‐yl)‐1,3‐dioxoisoindolin‐4‐yl)oxy)acetamido)ethoxy)ethoxy)ethyl)benzamide (MC4387, 2)

Red solid; m.p.: 140–142 °C; recrystallization solvent: toluene; yield: 40%; ^1^H‐NMR (400 MHz, CDCl_3_) *δ* 2.08–2.12 (m, 1 H, NCHC*H*HCH_2_CO glutarimide), 2.27 (s, 3 H, CH_3_), 2.32 (s, 3 H, CH_3_), 2.33 (s, 3 H, CH_3_), 2.60–2.86 (m, 3 H, NCHCH*H*C*H*_2_CO glutarimide), 3.38–3.44 (m, 2 H, NHCH_2_), 3.56–3.63 (m, 10 H, 5 × CH_2_ linker protons), 4.55 (s, 2 H, OCH_2_CO), 4.85–4.88 (m, 1 H, NCHCH_2_CH_2_CO glutarimide), 6.85 (d, 1 H, CH furan proton), 6.94 (br s, 1 H, CONH), 7.07 (d, 1 H, CH phthalimide proton), 7.20 (s, 1 H, CH methine proton), 7.44 (d, 1 H, C*H* phthalimide proton), 7.47 (s, 1 H, C*H* phenyl proton), 7.56 (br s, 1 H, CON*H*), 7.58–7.64 (m, 2 H, 1 × C*H* phenyl proton + 1 × CH phthalimide proton), 7.80 (d, 2 H, 2 × CH benzamide protons), 7.98 (d, 2 H, 2 × CH benzamide protons), 8.57 (br s, 1 H, NH glutarimide), 8.74 (d, 1 H, CH furan proton). Elemental analysis for C_18_H_23_N_3_O_3_S: Calculated % = C, 62.16; H, 4.99; N, 9.45. Found % = C, 62.27; H, 5.01; N, 9.39. MS (ESI) *m/z*: 890 [M + H]^+^.

##### Biological Evaluation: Western Blotting and Immunoprecipitation Experiments

Following compound treatment for the indicated time frame (4 to 24 h), cells were solubilized, and the protein content was determined using the BCA protein assay (Pierce Chemical Co). Lysates were fractionated by SDS–polyacrylamide gel electrophoresis (SDS‐PAGE). Membranes were incubated with anti‐CBP (CBP (D6C5) Rabbit mAb #7389, Cell Signaling Technology), anti‐p300 (Anti‐KAT3B/p300 antibody (3G230), Abcam) or anti‐GAPDH (GAPDH Monoclonal Antibody (FF26A), Thermo Fisher Scientific) antibodies overnight. This was followed by incubation with the appropriate horseradish peroxidase‐conjugated anti‐mouse or anti‐rabbit secondary antibodies (Cytiva Europe) for 1 h. Enhanced chemiluminescence detection was then done following the manufacturer's instructions. GAPDH was used as a loading control. Images were quantified with the internal software of the Fusion Solo S (Vilber).

##### Biological Evaluation: MTT Proliferation Assay

Lymphoma cell lines were exposed to a large range of concentrations of each compound as single agent for 24 h or 72 h, followed by MTT and after 4 h SDS to stop the reaction. The day after, plates were read using a Cytation 3 plate reader (Biotek) and IC50s were calculated. DMSO was used as a control (untreated). IC50 is defined as the drug concentration giving 50% of proliferating cells compared to vehicle treated cells. IC_50_ was calculated by fitting the values via nonlinear regression (GraphPad Prism 8.0). Values represent mean ± S.D.

## Conflict of Interest

The authors declare no conflict of interest.

## Supporting information

Supplementary Material

## Data Availability

The data that support the findings of this study are available from the corresponding author upon reasonable request.
