# Supplementary material for: Development of C646‐Based Proteolysis Targeting Chimeras Degraders of the Lysine Acetyltransferases CBP and p300
Source: ChemMedChem. 2025 Apr 11;20(12):e202400792. doi: 10.1002/cmdc.202400792 (PMC12174726; doi:10.1002/cmdc.202400792)
Supplement: Supplementary file 1 — Supplementary Material [file CMDC-20-e202400792-s001.pdf]

Development of C646-based PROTAC Degraders of the Lysine Acetyltransferases CBP and p300

Francesco Fiorentino,[a, †] Filippo Spriano, [b, †] Daniela Tomaselli,[a] Giorgia Risi,[b] Emanuele Fabbrizi,[a] Valeria Pecci,[c] Simona Nanni,[c,d] Francesco Bertoni,[b, e, *] Dante Rotili,[a, f, g, *] Antonello Mai[a]

[a] *Department of Drug Chemistry and Technologies, Sapienza University of Rome, P.le A. Moro 5, 00185 Rome, Italy.*

[b] *Institute of Oncology Research, Faculty of Biomedical Sciences, USI Bellinzona, Switzerland.*

[c] *Department of Translational Medicine and Surgery, Università Cattolica del Sacro Cuore, Rome, Italy.*

[d] *Fondazione "Policlinico Universitario A. Gemelli IRCCS", Rome, Italy.*

[e] *Oncology Institute of Southern Switzerland (IOSI), Ente Ospedaliero Cantonale Bellinzona, Switzerland.*

[f] *Department of Science, "Roma Tre" University, V.le G. Marconi 446, 00146 Rome, Italy.*

[g] *Biostructures and Biosystems National Institute (INBB), Via dei Carpegna 19, 00165 Rome, Italy.*

[†] Equal contribution

* Corresponding authors:

Dante Rotili - Phone: +39 06 49913237; Email: dante.rotili@uniroma1.it

Francesco Bertoni - Phone: +41 58 666 7206; Email: francesco.bertoni@ior.usi.ch

Table S1. Elemental analysis of compounds **1** and **2**.

Compd	Formula	Calculated, %			Found, %		
		C	H	N	C	H	N
1	$C_{27}H_{23}N_3O_3S$	62.49	5.45	8.75	62.60	5.46	8.70
2	$C_{18}H_{23}N_3O_3S$	62.16	4.99	9.45	62.27	5.01	9.39

Table S2. IC_{50} values (μM) of compounds **1** and **2** after 72h treatment in SU-DH-L10, SU-DHL-2, SU-DHL-16, TMD8, and WSU-DLCL2 cell lines.[a]

Cell Line	IC_{50} (μM)[b]	
	1	2
**SU-DHL-10**	2.73 ± 0.072	6.34 ± 1.76
**SU-DHL-2**	2.05 ± 0.068	4.96 ± 0.33
**SU-DHL-16**	2.99 ± 0.19	8.02 ± 2.46
**TMD8**	1.95 ± 0.069	7.81 ± 3.04
**WSU-DLCL2**	3.24 ± 0.28	5.23 ± 0.85

[a] Values are means ± standard deviation (SD) of two separate experiments (n = 2).
[b] Half maximal inhibitory concentration: dose required to reduce cell proliferation by 50%.

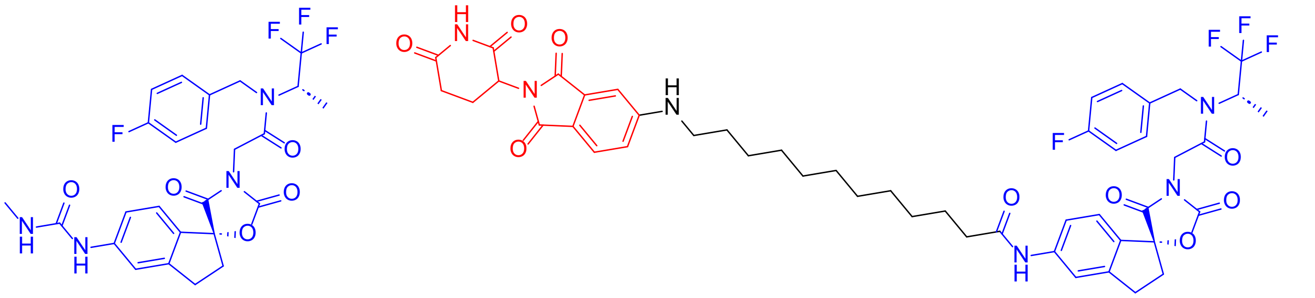

A-485 **JQAD1**

Figure S1. Chemical structures of A-485 and JQAD1.

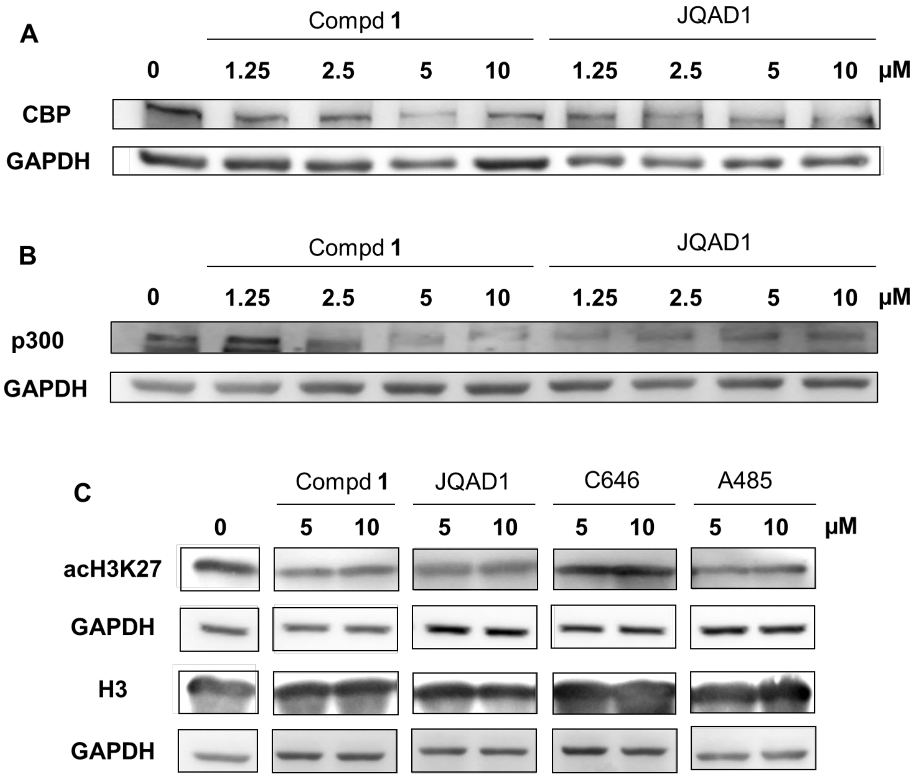

Figure S2. (A) WB analysis of CBP expression levels in the SU-DHL-10 cell line exposed to increasing concentrations of compound 1 and JQAD1 for 24 h (relative to Figure 3E). GAPDH has been used as a loading control. (B) WB analysis of CBP expression levels in the SU-DHL-10 cell line exposed to increasing concentrations of compound 1 and JQAD1 for 24 h (relative to Figure 3F). GAPDH has been used as a loading control. (C) WB analysis of H3K27ac levels in the SU-DHL-10 cell line exposed to compound 1, JQAD1, C646, and A485 (5 and 10 μM) for 24 h. The relative protein levels are expressed as a fold change of the H3K27ac/H3 ratio in treated versus untreated samples, after GAPDH normalization.
